# Morphometric analysis of tooth morphology among different malocclusion groups in a hispanic population

**DOI:** 10.1186/s12903-023-02882-7

**Published:** 2023-04-03

**Authors:** Hesham Alsaigh, Murad Alrashdi

**Affiliations:** 1 Orthodontics Section, Dentistry Administration, King Fahad Medical City, Riyadh, Saudi Arabia; 2 Department of Orthodontic and Pediatric Dentistry, College of Dentistry, Qassim University, Buraydah, Saudi Arabia

**Keywords:** Tooth morphology, Shape, Hispanic, Tooth size, Malocclusion

## Abstract

**Background:**

There have been reports of unique dental morphological features amongst Latin American and Hispanic populations, and this might invalidate the use of current orthodontic diagnostic tools within this population. There are no tooth size/tooth ratio normative standards for the Hispanic population, despite overwhelming evidence about differences in tooth size between racial groups.

**Objective:**

This study aimed to determine whether there are significant differences in 3-D tooth shape between patients with Angle Class I, Class II, and Class III dental malocclusion in the Hispanic population.

**Methodology:**

Orthodontic study models representing Hispanic orthodontic patients with Angle Class I, II, and III dental malocclusions scanned using an intra-oral scanner. The scanned models were digitized and transferred to a geometric morphometric system. Tooth size shape were determined, quantified, and visualized using contemporary geometric morphometric computational tools using MorphoJ software. General Procrustes Analysis (GPA) and canonical variates analysis (CVA) used to delineate the features of shape that are unique to each group.

**Result:**

The study revealed differences in tooth shape between the different dental malocclusion groups on all twenty-eight teeth that were studied; the pattern of shape differences varied between the teeth and the dental malocclusions. The MANOVA test criteria, F approximations, and P-values show that shape in all the groups was significantly different < 0.05.

**Conclusion:**

This study revealed differences in tooth shape between the different dental malocclusions on all teeth, and the pattern of shape differences varied between the different dental malocclusions group.

## Introduction

Morphological analysis of teeth is critical for the diagnosis and treatment of patients with a wide variety of oral and craniofacial pathologies. There are multiple ways that have been described for measuring teeth [1, 2]. However, the methods that are pertinent to clinical management are generally focused on the clinical crown. Traditionally dental morphometrics have used linear metrics such as mesiodistal, buccolingual, and occluso-gingival dimensions [3]. Historically, tooth morphology has been studied using manual techniques which involve a variety of calipers or the Boley gauge; instruments that can only obtain linear measurements [4–9]. These methods are limited to providing tooth size and are inherently incapable of detecting variations in tooth shape, form, and surface topography [10]. The establishment of more detailed methods has included identifying more landmarks on teeth, [11, 12] introducing angles within teeth, [13] and the use of occlusal polygons [14, 15]. A significant development was the combined use of high-definition photographs and computer technology [16]. Occlusal polygons were further incorporated into elliptical Fourier analysis (EFA) and used to analyze molar shapes [17]. Bernal (2007) used similar approaches but went further to subject the data to generalized Procrustes analysis (GPA) [10].

Geometric morphometric method (GMM) is increasing being applied to dental and craniofacial investigations [18]. Using aspects of GMM, Pavoni et al. evaluated the palatal morphology in children with impacted incisors, [19] Al Shaharani et al. evaluated the morphology of molars in patients with hypodontia [20] and Paoloni evaluated palatal morphology in Class II patients [21]. GMM has also been used to assess skeletal morphology in Class II and Class III and cleft lip and palate patients, [22–25] as well as to evaluate dental arch morphology of different malocclusion groups [26].

Advances in digital imaging and scanning have facilitated the recording of landmarks as coordinates. Robinson et al. used this concept to study tooth form from a photographic image using two-dimensional (x, y) coordinate; [27, 28] thus introducing a novel application in the study of tooth morphology. Their methodology used two-dimensional (2-D) data, as opposed to 3-D, and therefore provided only partial description of shape [29]. GMM has been used to study arch form, [30] tooth surface reconstruction, [31] and dental anthropology [32, 33].

Three-dimensional imaging has found applications in orthodontics [34]. Archives of 3-D orthodontics study models produce images that are identical to the original study models, easing access, study and exchange of clinical data [35–38].

There is no research investigating differences in tooth shape among the Hispanic population. The diagnosis and management of Tooth Size Discrepancy (TSD) for Hispanics has relied on standards that do not represent the group. The aim of this study was to determine whether there are significant differences in 3-D tooth shape between patients with Angle Class I, Class II, and Class III dental malocclusion, in the Hispanic population using GMM.

## Materials and methods

### Sample size and study sample

The sample size was determined based on previous studies which had used linear measurements as opposed to 3-D [39]. The sample size calculations showed that when two groups of 20 samples were compared a power of 80% power detected a 0.90 mm size difference. The total sample size was 120. Forty subjects in each group; 20 male and 20 female, with an age range between 12 and 55 years old. The subjects’ materials were obtained from the records of orthodontic patients at the graduate and faculty orthodontic clinics at University of Texas Health San Antonio, School of Dentistry. The study sample consisted of intra-oral scans (iTero scanner, Align Technologies, San Jose, CA) of Hispanic patients selected from the patient database. It included initial and final orthodontics study models of patients who had previous treatment. Successive cases that met the selection criteria were selected until the sample size was achieved. The following definitions and criteria were used to select subjects for the study groups: Group 1: Class I (ANB angle 0–4 degrees, Class I molar relationship), Group 2: Class II (ANB angle 4 or more degrees, Class II molar relationship), and Group 3: Class III (ANB − 1 or less degrees, Class III molar relationship).

#### Inclusion criteria and exclusion criteria

The study inclusion criteria were male and female participants from one demographic area (Southwest, Texas, USA) of Hispanic ethnicity and age between 12 and 55 years old with good quality orthodontics intraoral scan and no evident facial and dentoalveolar asymmetry.

Participants were excluded if their teeth were not fully recorded on the intraoral scan, had extensive dental restoration, had traumatized or severely worn teeth, or were patients with craniofacial anomalies.

### Scanning and landmarks

All the orthodontic study models were scanned with maximum resolution using an intra-oral scanner (iTero scanner, Align Technologies, San Jose, CA). The dental landmarks were tooth specific, where 19 points of the landmark were used for molars, 16 for premolars, and 12 for anterior teeth. Table 1 provide a list and definitions of all landmarks used for geometric morphometric analysis, 19 landmark for molars, 16 for premolars, and 12 for anterior teeth. The 3-D scanned models were saved in the STL format and identified by the same investigator using the software Checkpoint (Stratovan Corporation, Davis, CA). [40] The x, y, z coordinates defining the landmarks were exported as simple data text files and uploaded onto MorphoJ 1.07a. [41] a software designed to perform geometric morphometric analysis.

**Table 1:** Landmarks used for Geometric Morphometric Analysis

No.	Upper and Lower Molars	Upper and Lower Premolars	Upper and Lower Anterior Teeth
1	Mesial contact points	Mesial contact points	Mesial contact points
2	Distal contact points	Distal contact points	Distal contact points
3	Occlusal extent of buccal groove	Lingual cusp tip	Gingival margin lingual middle point
4	Occlusal extent of lingual groove	Buccal cusp tip	Incisal middle point
5	Mesial lingual cusp tip	Mesial point of buccal cusp	Mesial point incisal
6	Distal lingual cusp tip	Distal point of buccal cusp	Distal point incisal
7	Mesial buccal cusp tip	Mesial pit	Gingival margin buccal most mesial point
8	Distal buccal cusp tip	Distal pit	Gingival margin buccal most distal point
9	Central pit	Gingival margin buccal most mesial point	Gingival margin buccal middle point
10	Gingival margin buccal most mesial point	Gingival margin buccal most distal point	Middle point buccal
11	Gingival margin buccal most distal point	Gingival margin buccal middle point	Gingival margin lingual most mesial point
12	Gingival margin buccal middle point	Middle point buccal	Gingival margin lingual most distal point
13	Middle point buccal	Gingival margin lingual most mesial point	
14	Gingival margin lingual most mesial point	Gingival margin lingual most distal point	
15	Gingival margin lingual most distal point	Gingival margin lingual middle point	
16	Gingival margin lingual middle point	Middle point lingual	
17	Middle point lingual		
18	Distobuccal cusp tip (lower first molar)		
19	Occlusal extent of distobuccal groove (lower first molar)		

#### Geometric morphometric analysis

The landmarks’ x, y and z coordinates for each tooth were uploaded onto the software MorphoJ 1.07a. [41]. The first pre-analysis process was to detect outliers. MorphoJ 1.07a. provided an output comparing each specimen to the mean output of all individual specimen. The output also included a plotting of the distribution of specimen distances compared to the mean shape of all specimens in a group [41]. The extreme outliers were discarded. Previous work has attributed the extreme outliers to errors in instrumentation.

The individual outcomes were rotated, centered, and scaled to remove all non-shape related variations, using General Procrustes Analysis (GPA). [42, 43] This was followed by canonical variates analysis (CVA) using MorphoJ 1.07a to delineate the features of shape that are unique to each of the four groups. These were displayed as wireframe graphs which were used as the read-out for differences in morphological shape among the groups. A discriminant function analysis (DFA) was used to create wireframe graphs displaying the differences between any two groups.

#### Validation of landmark reproducibility

For intraobserver error, six permanent teeth measured: Lower left first molar, lower left canine, lower right second premolar, upper right first molar, upper right central incisor, and upper left first premolar. These teeth were identified on intra-oral scans obtained as described above. The specific tooth landmarks were obtained on each of the scanned images, and this was repeated three times with intervals of one week using Stratovan Checkpoint software by one examiner. The data was processed by a Procrustes ANOVA in MorphoJ 1.07a and the digitization error assessed.

## Results

Of the 183 sets of dental casts, 120 were analyzed. Forty subjects in each group; 20 male and 20 female in each group, with an age range between 12 and 55 years old. The other 63 dental castes were excluded due to either teeth were not fully recorded on the intraoral scan, presence of extensive dental restorations, traumatized or severely worn teeth, or patient with craniofacial anomalies. The result of the intraobserver analysis showed an excellent landmark reproducibility, where the mean squares (MS) of shape digitization errors were smaller than MS of individuals, Table 2.

**Table 2:** Digitization Error of Shape

Tooth#	Effect	Error of Shape								
		Individual					Digitization Error			
		SS	MS	DF	F	P		SS	MS	DF
upper right first molar		0.38238947	0.0009656300	396	36.40	< 0.0001		0.02334273	0.0000265258	880
upper right central incisor		0.92324788	0.0035373482	261	13.36	< 0.0001		0.15354333	0.0000337027	580
upper left first premolar		0.45683295	0.0012380297	369	36.73	< 0.0001		0.02763624	0.000038	820
lower left first molar		1.01743469	0.0022609660	450	132.70	< 0.0001		0.01703879	0.0000170388	1000
lower left canine		2.39298744	0.0091685342	261	26.99	< 0.0001		0.19702193	0.0003396930	580
lower right second premolar		1.08931960	0.0029520856	369	94.50	< 0.0001		0.02561479	0.0000312375	820

**Fig. 1:**
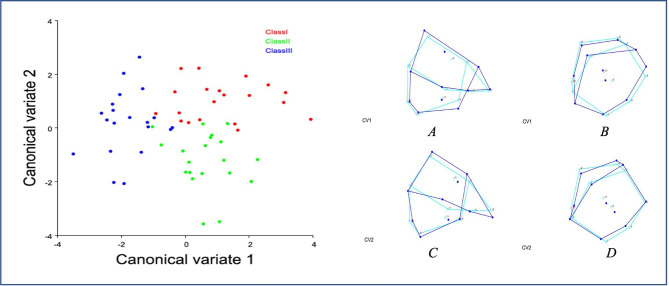
Left side - Scatter Plots of Principal Components CV1 and CV2 of Shape Variables in The Right Lateral Incisors. Right side - Shape Changes in The Right Lateral Incisors Displayed in Wireframe Graphs from Discriminant Function Analysis (DFA) between Each Two Groups of Malocclusion. Light Blue: Average Configuration of All Individuals. Dark Blue: Change on the Positive Axis in Mahalanobis Distance Units

The multivariate analysis of variance test criteria, F approximations, and P-values for the hypothesis of no overall malocclusion effect using Wilks’s Lambda test showed that shape in all the groups was significantly different for all teeth (Tables 3 and 4). The changes in shape are displayed in wireframe graphs associated with each CV, where the light blue representing the mean configuration of all the individual shapes and the dark blue determines a 5 Mahalanobis distance units change. Figure 1 shows an example of upper lateral incisors between subjects with Angle Class I, II, and III malocclusions. The results yielded a significant difference in the shape of the right and left lateral incisors when compared between groups of different dental malocclusion, where the first canonical (CV1) explains 77.59% of the total variation, followed by 22.40% for the second canonical (CV2). CV1 separates Class II (positive axis) from Class I and III (negative axis). While CV2 separates Class I and Class II (positive axis) from Class III (negative axis).

**Table 3:** MANOVA Test Criteria and F Approximations for the Hypothesis of No Overall Malocclusion Effect using Wilks’s Lambda for upper Permanent teeth

Statistic	Value	F Value	Num DF	Den DF	Pr > F
Upper right second molars	0.57674501	2.41	20	152	0.0014
Upper right first molars	0.52859224	3.79	20	202	< 0.0001
Upper right second premolars	0.45628836	4.47	20	186	< 0.0001
Upper right first premolars	0.51748010	3.94	20	202	< 0.0001
Upper right canines	0.68827764	1.70	20	166	0.0369
Upper right lateral incisors	0.57883233	2.80	20	178	0.0002
Upper right central incisors	0.64148491	2.24	20	180	0.0029
Upper left central incisors	0.69620727	2.00	20	202	0.0085
Upper left lateral incisors	0.62971370	2.37	20	182	0.0015
Upper left canines	0.58521175	2.64	20	172	0.0004
Upper left first premolars	0.59689648	2.91	20	198	< 0.0001
Upper left second premolars	0.45628836	4.47	20	186	< 0.0001
Upper left first molars	0.61148273	2.68	20	192	0.0003
Upper left second molars	0.66878648	1.60	20	144	0.0590

**Table 4:** MANOVA Test Criteria and F Approximations for the Hypothesis of No Overall Malocclusion Effect using Wilks’s Lambda for lower Permanent teeth

Statistic	Value	F Value	Num DF	Den DF	Pr > F
Lower left second molars	0.50355871	2.74	20	134	0.0003
Lower left first molars	0.38033365	5.53	20	178	< 0.0001
Lower left second premolars	0.42985192	5.04	20	192	< 0.0001
Lower left first premolars	0.41738604	4.99	20	182	< 0.0001
Lower left canines	0.60909834	2.11	20	150	0.0060
Lower left lateral incisors	0.66541502	2.10	20	186	0.0055
Lower left central incisors	0.74419686	1.54	20	194	0.0705
Lower right central incisors	0.64206521	2.55	20	206	0.0005
Lower right lateral incisors	0.57122396	2.91	20	180	< 0.0001
Lower right canines	0.50307833	3.24	20	158	< 0.0001
Lower right first premolars	0.58449710	2.65	20	172	0.0004
Lower right second premolars	0.53326385	3.25	20	176	< 0.0001
Lower right first molars	0.54459802	3.37	20	190	< 0.0001
Lower right second molars	0.38576124	3.42	20	112	< 0.0001

Discriminant function analysis (DFA) generated comparative a wireframe graphs between each two groups. Among the three malocclusion groups, the most morphological difference was between Class I and Class II groups for the following teeth: Upper right second molars, Upper right first molars, Upper right second premolars, Upper right first premolars, Upper right canines, Upper right lateral incisors, Upper right central incisors, Upper left lateral incisors, Upper left canines, Upper left canines, Upper left second premolars, Upper left first molars. Lower left first molars, Lower left second premolars, Lower left lateral incisors, Lower left central incisors, Lower right second premolars, Lower right second molars.

Another morphological difference was noted between Class I and Class III groups for upper left central incisors, and Lower right central incisors. For Class II and Class III groups, morphological difference noted in upper left second molars, lower left second molars, lower left first premolars, lower left canines, lower right lateral incisors, lower right canines, lower right first premolars, and lower right first molars. Tables 5 and 6 showed details description of the Mahalanobis Distances from Conical Variates Analysis for upper and lower Permanent dentition.

**Table 5:** Mahalanobis Distances from Conical Variates Analysis for Upper Permanent Teeth

	Group	Class I	Class II	Class III
Upper right second molars	Class I	-	< 0.0001	< 0.0001
	Class II	3.80	-	< 0.0001
	Class III	2.95	3.53	-
Upper right first molars	Class I	-	< 0.0001	< 0.0001
	Class II	3.05	-	< 0.0001
	Class III	3.03	3.03	-
Upper right second premolars	Class I	-	< 0.0001	< 0.0001
	Class II	3.56	-	< 0.0001
	Class III	3.49	3.47	-
Upper right first premolars	Class I	-	< 0.0001	< 0.0001
	Class II	3.06	-	< 0.0001
	Class III	2.54	2.70	-
Upper right canines	Class I	-	< 0.0001	< 0.0001
	Class II	2.22	-	0.0020
	Class III	2.03	1.74	-
Upper right lateral incisors	Class I	-	< 0.0001	< 0.0001
	Class II	3.20	-	< 0.0001
	Class III	1.94	2.36	-
Upper right central incisors	Class I	-	< 0.0001	< 0.0001
	Class II	2.58	-	< 0.0001
	Class III	2.43	2.06	-
Upper left central incisors	Class I	-	< 0.0001	< 0.0001
	Class II	2.10	-	0.0001
	Class III	2.17	1.73	-
Upper left lateral incisors	Class I	-	< 0.0001	< 0.0001
	Class II	2.40	-	0.0003
	Class III	2.05	1.79	-
Upper left canines	Class I	-	< 0.0001	0.0002
	Class II	2.22	-	< 0.0001
	Class III	1.93	2.13	-
Upper left first premolars	Class I	-	< 0.0001	< 0.0001
	Class II	3.20	-	< 0.0001
	Class III	2.65	2.40	-
Upper left second premolars	Class I	-	< 0.0001	< 0.0001
	Class II	3.24	-	< 0.0001
	Class III	2.74	2.86	-
Upper left first molars	Class I	-	< 0.0001	< 0.0001
	Class II	3.39	-	< 0.0001
	Class III	2.67	2.68	-
Upper left second molars	Class I	-	< 0.0001	< 0.0001
	Class II	2.77	-	< 0.0001
	Class III	2.43	2.93	-

**Table 6:** Mahalanobis Distances from Conical Variates Analysis for Lower Permanent Teeth

Teeth	Group	Class I	Class II	Class III
Lower left second molars	Class I	-	< 0.0001	< 0.0001
	Class II	3.49	-	< 0.0001
	Class III	3.63	4.02	-
Lower left first molars	Class I	-	< 0.0001	< 0.0001
	Class II	5.16	-	< 0.0001
	Class III	4.63	4.56	-
Lower left second premolars	Class I	-	< 0.0001	< 0.0001
	Class II	2.85	-	< 0.0001
	Class III	2.78	3.21	-
Lower left first premolars	Class I	-	< 0.0001	< 0.0001
	Class II	3.59	-	< 0.0001
	Class III	3.61	4.03	-
Lower left canines	Class I	-	< 0.0001	< 0.0001
	Class II	2.46	-	< 0.0001
	Class III	2.60	2.64	-
Lower left lateral incisors	Class I	-	< 0.0001	< 0.0001
	Class II	2.61	-	< 0.0001
	Class III	1.81	2.18	-
Lower left central incisors	Class I	-	< 0.0001	< 0.0001
	Class II	2.00	-	< 0.0001
	Class III	1.76	1.98	-
Lower right central incisors	Class I	-	< 0.0001	< 0.0001
	Class II	2.04	-	< 0.0001
	Class III	2.23	1.80	-
Lower right lateral incisors	Class I	-	< 0.0001	< 0.0001
	Class II	2.00	-	< 0.0001
	Class III	2.06	2.28	-
Lower right canines	Class I	-	< 0.0001	< 0.0001
	Class II	2.69	-	< 0.0001
	Class III	2.04	3.57	-
Lower right first premolars	Class I	-	< 0.0001	< 0.0001
	Class II	2.17	-	< 0.0001
	Class III	3.01	3.11	-
Lower right second premolars	Class I	-	< 0.0001	< 0.0001
	Class II	2.33	-	< 0.0001
	Class III	3.26	3.21	-
Lower right first molars	Class I	-	< 0.0001	< 0.0001
	Class II	2.67	-	< 0.0001
	Class III	3.32	3.72	-
Lower right second molars	Class I	-	< 0.0001	< 0.0001
	Class II	4.28	-	< 0.0001
	Class III	4.09	3.76	-

## Discussion

The GMM methods that were used in this study ensured detailed and objective quantification of the shape of the study samples. In addition, the GMM methods circumvented the inability of traditional metric and angular analyses to separate the effects of size on shape [10, 18, 44, 45]. The relatively large number of landmarks and their optimal distribution facilitated the capture of enough data; thus, ensuring accuracy in describing shape. This contrasts to the techniques which have been used variously to quantify tooth shape. Most of these are derived from traditional linear measurements, especially the ratio of mesio-distal (MD) and bucco-lingual (BL) metrics [46, 47]. The information derived is limited and does not describe most of the in tooth shape. The adaptation of 3D GMM analysis of subjects who fit into three subgroups of malocclusion was not only more efficient, but also more reproducible.

Differences in tooth shape between various racial groups have been reported. Lavelle (1972) studied the dental crown diameters of a White, African American, and Southeast Asian population sample [48]. The study reported the smallest dimensions in the White sample, next was the Asian sample, and the African American subjects had the largest dimension. Merz et al. (1991) reported similar findings, with larger mesio-distal canine, premolar and molar crown dimensions of the African American subjects [49]. According to Yuen et al. (1997), Australian Aboriginals had larger tooth dimensions compared to the Hong Kong Southern Chinese population, and Caucasians who had the smallest dimensions of the populations studied [50]. In a study comparing the mesio-distal tooth width of White British males to British of Pakistani origin, Radnzic (1987) found no statistically significant differences between the groups; concluding that the populations may have shared a common Caucasian lineage [51]. Other studies concluded that the population in Iceland had larger tooth dimensions compared to other Europeans [52]. In addition, Brook et al. (2009) reported that a Southern Chinese population had the largest mesio-distal crown size compared to a White North American population sample, and the Romano British sample had the smallest dimensions in the study [53].

Tooth shape is influenced by several factors [54] These include genetic, epigenetic, and environmental, factors as well as evolutionary adaptation processes [55, 56]. In this study, tooth shape difference amongst teeth in dental malocclusions Class I, II, and III was reported for all the twenty-eight teeth that were analyzed. This contrasts to the difference in centroid size (CS) in the same sample; CS difference was detected in only four teeth. The differences in shape were very similar in principal and symmetrical between left and right. The shape changes were reproducible when analyzed and visualized using wireframes, and scatter plot of the first two conical variates graphs representing a change in Mahalanobis distance units. This finding was unusual from a basic tooth development perspective. Although tooth development is controlled by common morphogenetic pathways, each tooth germ develops as an independent biological entity [57].

The effects of dental malocclusion class on tooth shape in this study can be represented by the upper lateral incisor; a tooth that has been reported to contribute to TSD [58]. The results indicate did not show any significant malocclusion-related difference between the upper lateral incisors in both male and females samples. However, there were significant differences in the shape of lateral incisors among the different malocclusion groups. Buccal views of wireframe graphs show that Class I is wider in shape mesiodistally, and Class II is longer in shape. The shape of the lateral incisor in Class III malocclusion did not show any significant difference from either Class I or II.

The effects on shape reported for the upper lateral incisor in this study are not in conformity with what would have been expected based on previous studies. Benward et al. [58] reported a higher level of tooth deformities in the maxilla of Class III patients. Eustaquio Araujo [59] concluded that the anterior tooth size discrepancy was greater in Class III patients compared to Class I and Class II. In these studies, the maxillary discrepancies in Class III were attributed to the upper lateral incisor. Although it is conceivable that a larger sample size might produce results that would more closely reflect the previous studies, the dissimilarity may be due to differences attributable to the ethnic background of the population studied.

This study revealed differences in tooth shape between the different dental malocclusions on all twenty-eight teeth that were studied, and the pattern of shape differences varied between the dental malocclusions. In addition, the study showed some unique differences in shape of teeth compared to the more commonly studied population as exemplified by the upper lateral incisors. This suggests that the shape variation described is a unique entity inherent in the Hispanic population studied. This study provides some guidelines towards future directions. This includes using larger sample sizes, comparative population studies, and correlation with dental and craniofacial abnormalities, which was a limitation in this study.

## Conclusion

The shape variation is a distinct entity inherent in the Hispanic population, where there is a significant difference in 3-D tooth shape between patients with Angle Class I, Class II, and Class III dental malocclusions compared to other populations.

## Data Availability

All data generated during this study are included in this published article.
